# A human rights-based framework to assess gender equality in health systems: the example of Zika virus in the Americas

**DOI:** 10.1080/16549716.2019.1570645

**Published:** 2019-03-20

**Authors:** Carol Vlassoff, Ronald St. John

**Affiliations:** a School of Epidemiology and Public Health, University of Ottawa, Ottawa, Canada; b Centre for Emergency Preparedness and Response, Public Health Agency of Canada, Ottawa, Canada

**Keywords:** Gender and Health Inequality, Gender equality, health systems building blocks, human rights, Zika virus, Latin America and Caribbean

## Abstract

**Background**: The right to health was enshrined in the constitution of the World Health Organization (WHO) in 1946 and in the Universal Declaration of Human Rights in 1948. The latter Declaration, which also guaranteed women’s rights, was signed by almost all countries in the world. Subsequent international conventions reinforced these rights, requiring that women be able to realize their fundamental freedoms and dignity. Although the value of incorporating gender into health systems has been increasingly acknowledged over the years, gender inequalities in health persist.

**Objective**: To introduce a tool to help countries assess their performance in addressing gender inequalities in their health systems, using the example of the Zika virus (ZIKV) in countries of the Americas.

**Methods**: This paper is based on comprehensive reviews of the literature on the links between gender equality, health systems and human rights, and available scientific evidence about an adequate response to ZIKV.

**Results**: The authors present a simple two-part framework from the human rights perspectives of the health system as duty bearer, incorporating WHO’s six health system building blocks, and of its clients as rights holders. The authors apply the framework to ZIKV in the Americas, and identify strengths and weaknesses at every level of the health system. They find that when considering gender, health systems have focused mainly on dichotomous sex differences, failing to consider broader gender relations and processes affecting access to services, quality of care, and health outcomes.

**Conclusions**: The authors’ framework will permit countries to assess progress toward gender equality in health, within the context of their human rights commitments, by examining each health system building block, and the degree to which clients are realizing their rights. By applying the framework to specific health conditions, gender-related achievements and shortcomings can be identified in each health system component, fostering a more comprehensive and gender-sensitive response.

## Background

‘The right to enjoy the highest attainable standard of physical and mental health’ was enshrined in the constitution of the World Health Organization (WHO) in 1946. It was further elaborated in the Universal Declaration of Human Rights of the United Nations (UN) in 1948, which also guaranteed women’s rights. The Declaration was signed by almost every country in the world. Subsequent international conferences and conventions reinforced these rights, especially those related to sexual and reproductive health, requiring that women be protected from any interference in their fundamental freedoms and human dignity. In 1979, the UN General Assembly adopted the Convention on the Elimination of All Forms of Discrimination against Women (CEDAW), which defined the principle of equality between men and women, now referred to as ‘gender equality’, prohibited discrimination between the sexes, and provided an agenda to end it.

Gender refers to the socially constructed characteristics of women, men and others who do not fit into binary male or female sex categories [1], while gender equality in health means that people of all sexual orientations experience the same conditions and opportunities to realize their full rights and potential to be healthy [1]. All human beings are equally entitled to these ‘whatever our nationality, place of residence, sex, national or ethnic origin, colour, religion, language, or any other status’ [2]. In 1995, 171 of the UN member states reaffirmed their commitment to gender equality at the 4th World Conference on Women in Beijing [3], and by 2015, 189 of them had ratified or acceded to CEDAW [4]. Despite this commitment, there has been a general tendency, particularly in the fields of health and medicine, to see gender in binary terms – masculine and feminine – and to focus on differences between them in terms of external factors that determine their roles in society. Connell has argued that international institutions and gender scholars have perpetuated this static thinking, generally ignoring gender relations which involve many people and different categories, bodies and institutions [5,6]. She notes that ‘[g]ender is, above all, a pattern of social relations in which the positions of women and men are defined, the cultural meanings of being a man and a woman are negotiated, and their trajectories through life are mapped out. Gender relations are found in all spheres of life, including organizations’ [7,p.839]. People and institutions are ‘gendered’ in many ways [5–7], and an understanding of the relationships and power dimensions among and between them is central to the concepts discussed here.

**Figure 1.**
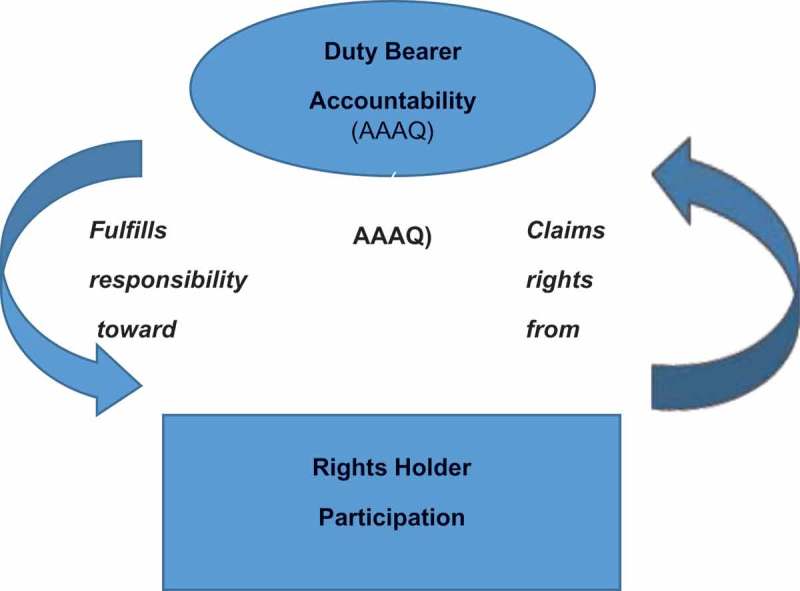
The reciprocal relationship between the duty bearer and rights holders.^6^

Within the international legal system, governments commit to respecting, protecting and fulfilling human rights. The right to health tasks health systems (the duty bearer) to assure the highest obtainable standard of health for their populations (the right holders), regardless of gender or other attributes (Figure 1). This standard is assessed by four benchmarks: availability, accessibility, acceptability and quality (AAAQ) [8]. Duty bearers are legally bound to fulfill their responsibilities toward rights holders, while rights holders are beholden to know and claim their freedoms and entitlements. Some rights (core obligations) have ‘immediate effect’, while others, for states not immediately able to comply with all requirements, have the option of ‘progressive realization’, i.e. providing some benefits incrementally, while moving steadily forward to fulfill all AAAQ-compliant benefits to the maximum of their resources [9].

The right to health contains five core obligations subject to immediate effect. Three are the sole responsibility of the health sector – access to discrimination-free health facilities, goods and services; access to essential drugs; and the equitable distribution of all health facilities, goods and services. The other core obligations – access to nutritious food and to safe and potable water – are shared with other sectors. From the client perspective, the right to health consists of receiving the above three components from the health sector in a non-discriminatory and supporting environment.

Despite the attention to gender, health and rights in the global development rhetoric, their systematic consideration and inclusion in health systems and the delivery of health care have been fragmented at best, and often completely lacking [10]. Typically, treatment of illness and disease is seen as the first priority, while contributing factors such as gender and human rights are postponed for later, if considered at all [11–13]. This is due, at least partly, to the separation of disciplines, health care being largely relegated to the biomedical sciences and its determinants and consequences, including gender and human rights, to the social sciences [14].

Efforts to address this disconnect, usually spearheaded by gender and human rights researchers and activists, have included the generation of a large body of evidence from qualitative and quantitative studies demonstrating that gender inequality, of which human rights inequities are inherent by definition, produces poorer health outcomes at the individual level [15], as well as inferior health systems performance [16,17]. These findings have spurred the development of elaborate training materials, workshops, and interdisciplinary courses for health and social science professionals [18,19]. However, according to evaluations, the impact of such approaches remains limited, and reference materials, such as manuals and guidelines, are rarely used in practice, especially in the public sector [13,14]. This seems to be due to the many competing priorities of health workers, their perception that the interventions involve additional work beyond the scope of their duties, and the lack of incentives and rewards for using these programs. Moreover, the materials, while grounded in extensive research and testing, are so detailed and exhaustive that they require considerable time and effort to be used by already overburdened health systems. For example, WHO (2011) [10] requires detailed assessments at three levels: a review of state obligations and commitments to human rights and gender equality, legal, policy and institutional frameworks for promoting human rights and gender equality, and an analysis of health sector strategies. However, this manual may be a useful reference document for implementing the current framework, especially for helping formulate questions for further analysis and discussion. A different approach for incorporating gender equality considerations into health systems seems to be needed, one that does not appear as an artificial add-on, but rather as a valuable component of an integrated whole leading to improved processes and outcomes.

In this paper we propose a framework to facilitate the incorporation of gender equality into health systems, using a human rights approach. It is intended to help countries assess the achievements and shortcomings of their health systems in honoring their international commitments to human rights and gender equality. Two important features of this framework are its simplicity and flexibility: it requires only an understanding of the main human rights obligations to which countries are already committed, a basic understanding of health systems and health conditions, and it is applicable to any health condition or geographical context. It incorporates both health system and client perspectives, facilitated by an openness on both sides to greater inclusivity, communication and participation.

To illustrate our framework, we have chosen the example of the Zika virus (ZIKV) in Latin America and the Caribbean (LAC) because it aptly captures the intersection between human rights, gender equality and health. ZIKV has a particular impact on vulnerable populations, especially poor women and their newborn infants, and for this reason our analysis, although including all gender groups, focuses more on women. However, it also recognizes that they are often in active relations with men, boys, and people of different sexual orientations, and these relationships help to determine access to, and interactions with, health staff and institutions, as well as health outcomes. Moreover, the framework may be applied to virtually any health problem which may affect males more than females, such as injuries and accidents, or gay and bisexual populations, in the case of HIV, for example. In the latter case, gender relations are highly relevant, due to the often greater vulnerability of the sexual partners of dominant males, as well as the negative or positive attitudes of friends, family members, and health workers, either exacerbating or assuaging the suffering and responses of those affected.

### Gender equality and the right to health: ZIKV in the Americas

ZIKV is transmitted primarily through the bite of infected *Aedes* (*aegypti* and *albopictus*) mosquitoes. In adults, infection may be followed by Guillain–Barre syndrome (GBS), an autoimmune neurologic condition [20]. ZIKV can be vertically transmitted during pregnancy, and may result in Congenital Zika Syndrome (CZS), which includes microcephaly and neurological brain damage in newborns. The probability of ZIKV infection affecting a pregnancy or fetus remains unknown, and may be as low as 1%. No cases of mother to child transmission through breastfeeding have been documented and no adverse neurological outcomes have been reported in infants with postnatally acquired ZIKV disease in countries with ongoing transmission of ZIKV [21]. WHO advises that the benefits of breastfeeding for both infant and mother outweigh any potential risk of ZIKV transmission through breastmilk. However, the virus can be transmitted through sexual contact if one of the partners is infected [20].

There is currently no vaccine to prevent infection. Diagnosis is difficult because tests for ZIKV can cross-react with some other viruses such as yellow fever and dengue [20]. There are many unknown factors associated with ZIKV infection, such as the probability of long-term neurological sequelae, the probability of developing GBS or other neurological complications, and the issue of sexual transmission through semen and female genital tract secretions, even after the virus can no longer be detected in blood or urine [22].

The virus targets people who live in conditions favoring mosquito breeding, such as precarious housing without sewage systems and regular water supply [11]. In order to prevent infection, comprehensive reproductive health services are necessary, including information on the availability of contraception, and in the case of pregnant women the possibility of abortion. However, in LAC, many pregnant women and their families lack adequate information about the risks of infection, microcephaly and its possible consequences, and how to access an abortion, where legal [23]. Moreover, the decision to undergo an abortion is highly dependent upon gender relations in the household and society (e.g. religious affiliations), as well as positive or negative interactions with health staff and others. Thus, as Rao observes, ‘The Zika virus outbreak is exposing the tragic failures of reproductive health and rights policies in Latin America’ [23, p. 842].

In LAC, the countries most affected by ZIKV have widely differing laws on women’s sexual and reproductive rights affecting prevention of infection. Restrictive abortion laws in many LAC countries leave pregnant women choosing to terminate their pregnancies with little choice but clandestine procedures. Several less severely affected countries – Cuba, Guyana, French Guiana, Uruguay, and Mexico – allow abortion without restriction, whereas others, including Chile, Haiti, Dominican Republic, El Salvador, Nicaragua, and Honduras, prohibit abortion altogether. Some countries, including Brazil, sanction abortion in exceptional circumstances – to save a woman’s life, rape, or if the fetus has anencephaly, a serious brain disorder [24]. In El Salvador laws against contraception and abortion are extremely severe, and women accessing these measures can be criminalized and imprisoned. However, even where contraception is encouraged by governments to avoid pregnancy in ZIKV endemic areas, uptake may be hindered by gender constraints. In Colombia, for example, a study of contraceptive use before and after a government-issued advisory encouraging women to delay childbearing due to possible ZIKV-related birth defects and permitting access to abortion in the case of infection found no increase in uptake of either option following the advisory [25]. The authors attributed this to cultural barriers and gender inequality, including women’s lack of autonomy in sexual and reproductive matters, inadequate sex education, and limited contraceptive access. Gender relations with partners and others also play an important role in women’s decision-making and access to abortion services. Overall, however, abortions appear to be on the rise in LAC: in 2010–2014, an estimated 6.5 million abortions were recorded, up from an estimated 4.4 million in 1990–2004 [26], representing the highest estimated annual rate among the world’s sub-regions [26]. This indicates that religious and cultural concerns may not be overwhelming obstacles to abortion access where it is legal and safe.

Gender inequalities influence women’s treatment options for children infected with ZIKV. In Brazil’s poorest and most affected regions, the north and north-east, women lack access to reliable contraception, medical care, and other basic necessities of life [12]. Day-to-day struggles, including government office closures, long waiting times, and failure of the government to honor its promise of financial assistance for infants with microcephaly, have been reported in Brazil’s northern region, home to 80% of microcephaly cases. Women bear the greatest burden in these situations, frequently including sole responsibility for child care, loss of employment, and abandonment by fathers. Moreover, therapeutic facilities, day care, and schooling opportunities are often inaccessible to them [27].

Despite these realities, there may be missed opportunities that could be better used to improve prevention, care and treatment for vulnerable populations. For example, there is potential for a greater contribution of men in combating ZIKV, not just in practicing safer sex, using condoms, and postponing conception, the role typically accorded to them [28]. Although women bear the children, men are equally important household members, as husbands, brothers, fathers and elders, and are often even more central when it comes to decision-making about expenditures, taking family members for health care, and caring for sick children when the mother is overburdened. Hence, men should be recognized as key partners in the ZIKV response, both by society and the health system [28].

Overall, health systems in LAC have tended to focus on the strengthening of laboratory and surveillance networks, rather than reaching out to populations most vulnerable to ZIKV and its consequences. Even where reproductive health services have been augmented, such as in Puerto Rico, other necessary services, such as physical therapy, have been neglected [11]. These shortcomings highlight the lack of holistic, gender-sensitive health system responses to the challenges of ZIKV in affected LAC countries which would understand gender as ‘multidimensional: embracing at the same time economic relations, power relations, affective relations and symbolic relations; and operating simultaneously at intrapersonal, interpersonal, institutional and society-wide levels’ [6, p. 1677].

## Methods

This paper is based on comprehensive reviews of the literature (over a period of approximately 18 months) on the links between gender equality, health systems and human rights, and available scientific evidence concerning an adequate response to ZIKV, in terms of prevention, counselling, care and treatment. Data were obtained from official statistics and published and unpublished national studies. Our search terms are those mentioned above, separately and combined (see Table 1). Out of a total of 295 sources identified, we excluded those which did not add significant new information to that already gathered. We conducted an in-depth review of the most relevant and authoritative peer-reviewed papers and documents, 169 in total. The literature on gender inequality in health (including prevention, access to services, adherence, determinants and consequences) and that concerning gender and human rights is far more abundant than that on gender and health systems (where reproductive rights have received most attention). We also searched for frameworks linking at least two of our main areas of interest (gender equality, health systems and human rights) (see Table 1), and found five that were particularly relevant [10,29–32] to the present analysis.

**Table 1.** Details of the sources reviewed.

Main search term(s)	Number searched	Number selected/reviewed	Key documents (full references in bibliography)
Gender inequality in health	84	51	Muralidharan et al. [18] Transforming gender norms, roles, and power dynamics for better health: evidence from a systematic review of gender-integrated health programs in low- and middle-income countries
Gender and health systems	22	12	Percival et al. [13] Health systems and gender in post-conflict contexts: building back better?
Gender and primary health care	10	7	WHO (31) Gender, women and primary health care renewal. A discussion paper
Gender and human rights	80	45	Nordic Trust Fund and World Bank (2013) Report of gender and human rights-based approaches in development
Gender, health, and human rights	13	7	WHO [10] Human rights and gender equality in health sector strategies: how to assess policy coherence WHO [1] Gender, equity and human rights
Human rights	8	5	United Nations Development Group (2003) The human rights based approach to development cooperation towards a common understanding among UN agencies
Right to health	15	12	Office of the High Commissioner for Human Rights [8] CESCR General Comment No. 14: the right to the highest attainable standard of health (Art. 12)
ZIKV general	14	8	Centers for Disease Control and Prevention (n.d.) Clinical guidance for healthcare providers for prevention of sexual transmission of Zika virus
ZIKV, gender, and human rights	31	17	Pan American Health Organization. ZIKA ethics consultation: ethics guidance on key issues raised by the outbreak. 6–7 April 2015 Rao [23] ZIKV virus outbreak: reproductive health and rights in Latin America
Frameworks	14	5	WHO [10] Human rights and gender equality in health sector strategies: how to assess policy coherence

Based on this review of authoritative sources and WHO’s six health system building blocks [33], we developed a simple two-part framework reflecting the perspectives of the health system and its clients. We then systematically extended our framework to ZIKV in order to demonstrate its applicability to a real-world situation. Specifically, for ZIKV we reviewed 25 peer-reviewed sources. Our analysis also benefits from the expertise and experience in the area of ZIKV of one of the authors. While many useful frameworks exist on relevant elements of our framework, ours is the first (of which we are aware) to take a human rights approach to analyze health systems responsibilities and client rights from a gender perspective.

## Results

### The framework

The following analysis presents our framework from the perspectives of the health system (Table 2(a)) and that of its clients (Table 2(b)).

**Table 2.** (a) Framework (Part 1) to assess gender-related health systems’ obligations vis-à-vis ZIKV.

	Gender-related programmatic obligations			
	Prevention			Care and treatment
Health system (duty bearer) perspective	Pregnancy and childbirth	Sexual transmission	Counselling	CZS and autoimmune neurological conditions
1. Leadership and governance	-Policies ensuring women’s and partners’ access to relevant reproductive health** **(RH) options	-Policies ensuring access to protective options for at-risk sexually active people	-Policies ensuring women’s and partners’ access to confidential RH counselling	-Policies for appropriate, gender and culturally sensitive treatment (Rx) and care
	-Oversight mechanisms exist, with community input	-Relevant oversight mechanisms exist	-Policies ensuring access of at-risk sexually active people to gender and culturally sensitive confidential counselling	-Oversight mechanisms exist
			-Oversight mechanisms exist	
Obligation met (yes/no, comment)				
2. Health financing	-Affordable reproductive insecticide treated nets (ITNs) provided -Access to safe and affordable RH care, including MTP -Budgeting for chronic care related to ZIKV	-Access to safe and affordable contraceptive methods	-Affordable, confidential, gender and culturally sensitive counselling services provided by health system	-Comprehensive and high-quality care and Rx provided free of cost to those in need
Obligation met (yes/no, comment)				
3. Health workforce	-Health workers trained in appropriate prevention measures -Strengthened laboratory and surveillance capacity	Health workers trained in range of appropriate contraceptive methods	Counsellors trained in ZIKV prevention and related gender and cultural issues	Health workers trained in gender and culturally sensitive care and Rx
Obligation met (yes/no, comment)				
4. Health services	-Timely screening and diagnosis of asymptomatic pregnant women, testing for symptomatic pregnant women and testing for infants with possible CZV implemented in gender and culturally sensitive way -ZIKV-related ANC services integrated with detection and Rx of malaria, dengue, as appropriate -Strengthened laboratory and surveillance services -Safe abortion and post-abortion services provided	Guidelines implemented in gender and culturally sensitive way	-ANC counselling integrates information with HIV, malaria, dengue, as appropriate -Counselling provided. including on abortion, in gender and culturally sensitive way	Comprehensive and high-quality care and Rx, including abortion, provided in gender and culturally sensitive way
Obligation met (yes/no, comment)				
5. Medical products	-ITNs and appropriate insecticides available -Full range of RH products available, including for MTP	-Adequate supply of male and female condoms available	-ITNs and appropriate insecticides available for demonstration -Full range of contraceptives available for demonstration	-To extent possible, state-of-art Rx available (e.g. ventilators) for those infected and symptomatic, in gender and culturally sensitive way
Obligation met (yes/no, comment)				
6. Health information and research	-Data disaggregated by sex and social determinants relevant to ZIKV widely disseminated in appropriate languages and formats -Surveillance ongoing and adequate -Comprehensive, timely information on ZIKV risks and pregnancy prevention universally disseminated in gender and culturally sensitive way -Open access/data sharing platforms for ZIKV information	-Comprehensive information on ZIKV risks and prevention of sexual transmission widely disseminated in gender and culturally sensitive way -Surveillance ongoing and adequate -Research (if feasible) conducted with full consent, in gender and culturally sensitive way	-Comprehensive information on ZIKV risks imparted in gender and culturally sensitive way -Patient feedback sought on quality of care and Rx, and results shared with relevant health services	-Research on Rx supported, with attention to sex differences in effects -Information on research results disseminated in timely manner, in gender and culturally sensitive way -Patient feedback sought on quality of care and Rx, and results shared with relevant health services
Obligation met (yes/no, comment)				
(b) Framework (Part 2) to assess clients’ realization of gender-related health care rights.				
Client perspective (right held)	Benchmarks (Yes/no, comment)			
Obligations	Available	Accessible	Accessible	Quality
Access to discrimination-free health facilities, goods, andservices				
Access to essential drugs				
Equitable distribution of health facilities, goods, and services				

### Health system (duty bearer) and ZIKV

In this section, we identify the main gender-related programmatic obligations of the health sector, focusing on the key health system building blocks (Table 2(a)). These blocks are highlighted in the first sub-section below, ‘Prevention’, but are not repeated in subsequent sections. Using ZIKV as an example, we identify the elements of an appropriate response in the areas of prevention, counselling, care and treatment, which may be used by different levels and programs of the health system to evaluate how adequately they are meeting their gender-related human rights obligations.

#### Prevention

Prevention advice generally focuses on vector control (insecticide spraying, eliminating standing water, bed nets) and personal protective measures, such as insect repellents. However, as these measures may have a limited impact on viral transmission, we prefer to focus on proven effective methods for prevention of congenital syndromes such as Zika-induced microcephaly. These include: access to voluntary contraception, including emergency contraception, for both men and women; access to comprehensive maternal health services, including antenatal care (ANC); and support for the voluntary medical termination of pregnancy (MTP) for women known to be infected [20].

The first health system building block (‘BB’), leadership and governance (Table 2(a)), is particularly important for policies regarding ZIKV prevention in pregnancy and childbirth. Many ZIKV-affected countries in LAC are advising women not to become pregnant for between 6 and 12 months, until the epidemic phase of the disease ends [25]. Policies in this area ethically entail offering complete, accurate and timely information and respecting women’s right to choose ‘[c]omprehensive sexual and reproductive care [encompassing] family planning, maternal health, pre-natal testing, safe termination of pregnancy, counseling, and post-natal care services’ [20,p.7]. Policies also should recognize the social context ) in which women’s decision-making occurs, their ability to make choices and act upon them. Advice should also include information for men, relevant family members, and people of different sexual orientations about risks and key recommended actions. For women infected during pregnancy, testing and comprehensive antenatal care and supportive social and health systems are essential to detect fetal abnormalities.

The reality of sexual transmission of ZIKV raises significant gender and human rights challenges for health systems. Universal access to safe and affordable medical products, including contraception, is especially important (2nd and 4th BB), and male and female condoms should be available to prevent sexual transmission (5th BB). Medical products should also include products necessary for MTP, where appropriate. Unfortunately, contraceptives are not universally available or affordable, making it difficult for men, women, and other genders to access them [31]. In many developing country contexts, women lack control over their bodies and are sometimes victims of non-consensual sex, sexual assault, and intimate partner violence [23,34,35]. As many as 56% of pregnancies in LAC are unintended [8]. In some cultures where the childbearing role of women is idealized, fear of social stigma may inhibit women from seeking family planning services [36]. Thus, for health workers, special efforts over and above their routine duties may be required to advocate for and facilitate full sexual and reproductive health for women with limited access to services, as well as to their partners (4th BB, Table (2a)), taking into consideration the complex social structures and gender relations in their communities. Strategies may include ‘mobile service delivery, expanded availability of emergency contraception, rural health worker deployment, community-based health promotion, and subsidized contraception … for the most vulnerable women’ [11,p.528], provided by health personnel in culturally and gender-sensitive ways (3rd BB). Where appropriate, ZIKV services may be integrated with related programs, including HIV, malaria, dengue, and Chikungunya.

As with the other prevention approaches, comprehensive information should be widely disseminated to women, men, and people of different sexual orientations (6th BB). A barrier to be addressed is the categorical view that family planning, pregnancy, and childbirth are primarily women’s concerns, and this perspective often prevails in health systems as well. Partly as a result, many cultural or religious norms prohibit or discourage open discussion of sexual issues among couples and even with health educators [28]. The HIV/AIDS pandemic has helped break down such barriers in the interests of public health [25] by, for example, involving religious leaders, peers, and educators, a lesson that could be applied to ZIKV as well.

Information and research obligations (6th BB) include widespread dissemination of information, in appropriate languages and gender and culturally acceptable formats, at all levels of the health system [24]. Ongoing surveillance and the provision of timely data, disaggregated by sex and relevant social determinants, are also supportive of prevention. Full information and informed consent must be assured for women asked to participate in clinical trials related to pregnancy or for post-mortem fetal examinations for research purposes. Men should also be involved in studies of natural history, infectivity, and clinical trials of vaccines and antiviral drugs [25].

#### Counselling

Policies to ensure that relevant health personnel are equipped to provide confidential and complete health counselling in gender and culturally sensitive ways are responsibilities of the duty bearer (Table 2(a)). For example, the Centers for Disease Control and Prevention (CDC) advise that pregnant women whose partners are exposed to ZIKV should be counselled to use condoms during every sexual encounter or avoid sex (of all kinds) with those partners for the duration of their pregnancies [37]. Given the cultural implications of this advice, and the often unequal gender relations which may prohibit women from discussing or negotiating sexual relationships, consideration should be given to involving both women and men, together or separately, in this counselling. Counsellors should be trained to take into account the socially embodied gender relations affecting their clients, especially those most at risk of infection, and tailor their advice accordingly. For instance, they should be aware that, while they cannot dictate clients’ behavior, they can at least equip them with the information needed to make an informed choice, and show that they are sensitive to their particular needs and situations. Counsellors should also try to integrate ZIKV and other relevant information for all genders, such as negotiation skills and the importance of efficient condom use for protection from HIV and sexually transmitted infections. For those who decide to bear a ZIKV-affected child, counsellors should be sensitive to the special needs of these women and couples, and referrals should be available for psychological and psychosocial counselling, disability care, and other social services. The involvement of both parents and other family members is especially critical in these cases [25]. Within the health care environment, counsellors are ideally placed to seek feedback from patients and community members about the performance of the health system and related services in order to improve quality.

#### Care and treatment

In the care and treatment component of our framework (Table 2(a)), we have grouped together the different health conditions thought to be associated with ZIKV and where appropriate health sector responses are still unclear.

Governments face new challenges in care and treatment in the ZIKV outbreak because diagnosis is difficult and because so little is known about the short- and long-term effects of infection. Other unknowns associated with ZIKV infection include the probability of long-term neurological sequelae or the probability of developing GBS, a condition that may follow infection in adults [20]. Lack of information makes surveillance, reporting and follow-up a critical public health function involving different levels of health services. It also poses counselling challenges for health workers faced with explaining these limited alternatives to vulnerable clients, who are forced to make difficult choices based on little knowledge and experience.

Policies are needed to provide for the appropriate treatment and care of those affected in a non-discriminatory and supportive environment, given that the virus is new, unfamiliar, and accompanied by considerable fear, uncertainty and stigma. Pregnant women and new mothers are particularly vulnerable in this respect. Given that effective treatment is still unavailable, health services should be adapted to incorporate the special counselling needs of their clients, with a focus on maternal child care, especially for adverse consequences.

Comprehensive and good quality care should be provided to clients free of cost. This is especially important for the poor in affected communities, among whom women predominate. Moreover, microcephaly-affected children will require concerted care over an uncertain lifetime, probably including multiple complicating conditions, entailing special attention as well for the mothers, fathers and other family members.

Research on treatments should be actively supported and promoted, with attention given to gender differences in their effects, and results should be disseminated in an understandable format, in a timely manner, and with attention to gender and cultural sensitivities.

### Clients (rights holders) and ZIKV

In this section we present the second part of our framework, referring to those human rights to which health system users are entitled under the right to health (Figure 1). We ask the question of how adequately clients in ZIKV-affected countries are receiving their minimum benefits from health systems in gender-appropriate ways (Table 2(b)). As Figure 1 shows, clients are responsible for claiming their rights through awareness and participation in their own health care. Making people aware of their rights, however, is a reciprocal responsibility of the health system.

The following assessment is limited by a paucity of client-focused social research on the impact of ZIKV, and by the lack of robust accountability mechanisms to compel governments to respond to clients’ claims [8].

#### Acc*ess to discrimination-free health facilities, goods and services*

The impact of ZIKV will be long-lasting for those affected, including for children with CZS and their care givers who will need ongoing access to appropriate health and social services. This may require reorganization of some health services to provide for these special needs, preferably with the involvement of their clients in how this should be done.

For clients to realize their rights, health services should provide AAAQ-compliant health facilities, goods and services, affordable for all, and information about appropriate prevention, care and treatment should be widely available in gender and culturally appropriate ways. These duties entail consideration of clients’ needs on the part of the services, including how clients can access them more easily. For example, could opening hours be adapted to times more convenient to users, both male and female? Could outreach services be provided to those with limited mobility, such as the poor or disabled? Poor women affected by ZIKV deserve special attention and should be consulted and counselled about the constraints they face (economic, health, transportation), the information they lack about ZIKV risks and prevention, and the stigma they may be experiencing due to the virus [11].

#### Access to essential drugs

Although there are no known drugs for the prevention of ZIKV transmission (with the exception of contraceptives and abstinence) or for its cure, clients have a right to affordable treatments to reduce suffering and pain. Diagnostic tests, and eventually treatments and perhaps a vaccine, should be available to all communities. An uninterrupted supply of contraceptive methods should be accessible to clients, as well as gender and culturally sensitive information on their use. As noted above, special efforts will be needed to combat social, economic, and gender barriers faced by ZIKV-affected populations in accessing appropriate family planning methods and advice. From a gender and human rights perspective, health systems should also ensure that products and facilities for MTP are available free of charge, although this remains a challenge in many political environments in LAC and elsewhere.

#### Equitable distribution of all health facilities, goods and services

Clients have a right to an equitable distribution of all health facilities, goods and services, regardless of gender, geographical, or other conditions. For example, facilities, goods and services are frequently concentrated in large cities; hence, rural clients, especially women and children, may not be accessing their right to health services. Within health services, distribution may be inequitable: personnel may favor some patients over others (e.g. men or better-off clients), or be more responsive to some illnesses or conditions than others [15]. Clients in all areas should be fully cognizant of their right to equitable health facilities, goods and services, within the context of their special gender needs, differences and relations.

## Discussions

The framework presented in this paper should be applicable to health systems in virtually any country which has ratified the Declaration of Human Rights. It can be used in the health system as a whole, or at different levels, as health systems are not homogenous and consist of many institutions and services. For example, it could be used in a single district, hospital, or community health facility, as relevant to existing mandates, opportunities, and constraints. It can also be applied, as in this paper, to specific programs within the health services. Prioritizing which actions to take will depend on the local situation, including religious and cultural contexts which dictate gender processes and relations, as well as societal norms and expectations. These are not generalizable to all countries or areas within countries. However, the human rights-based approach has the particular strength of being universal and inalienable, and health systems are bound to provide the best possible services to their clients, regardless of gender, cultural, religious, or other differences. Moreover, the rights-based approach allows for progressive realization, which gives countries the opportunity to move forward on their obligations at their own pace, hence allowing for contextual differences and adaptations, as long as they do not go backwards on their human rights commitments.

We have used ZIKV as an example because it presents obvious gender and human rights challenges, but the framework can equally well be applied to other diseases and health conditions. Consideration of both components (Table 2(a,b)) – the health system as duty bearer and the client as rights holder – and their intersectionality is essential. It is also important to keep in mind that health systems themselves are gendered, and the degree to which they are able to grasp complex gender concepts relating to their own work environment and to their clients will depend upon their particular contexts and training. In general, much more needs to be done before they are able to move beyond the dichotomous categorization of gender to a broader understanding of gender processes and relations.

We have given more attention to the health system perspective because understanding the clients’ needs and experiences is highly context-specific, and there is less evidence available on them in the literature. However, in applying the framework in country or sub-national contexts, equal attention should be paid to client participation to help evaluate the success of health services and to suggest improvements. A detailed guide to application of our framework is beyond the scope of this paper, and also requires the identification of context, health system needs, health issues being assessed, and prevailing gender relations. However, some of the key steps for implementation are provided in Table 3. Hopefully, this human rights-based framework to assess gender equality in health systems will help stimulate a more comprehensive approach to better incorporate gender and human rights considerations into health policies and programs, whether ongoing or in response to new public health crises.

**Table 3.** Example of implementation process.

Suggested steps	Participants/contributors
I. Obtain health system perspective	
1. Select health system component(s) (e.g. hospital, clinic, specialized service) and building block(s) to be assessed	Responsible personnel or task force identified for assessment (usually concerned health officials, researcher/evaluator)
2. Select health issue/condition, as relevant	Consult with biomedical specialists, concerned non-governmental organizations and civil society regarding health issue/condition and its impact
3. Garner expert advice on recommended policies and best practices	WHO and/or other relevant expert organizations; gender and human rights experts
4. Stakeholder consultations	Range of relevant stakeholders, including health staff, other relevant public and private sector stakeholders, and clients of all genders
II. Obtain client perspective	
1. Stakeholder interviews and consultations	Representatives of gender and human rights groups, and civil society, preferably of different genders, roughly representative of their distribution in the service area
2. Visit areas particularly affected by the weaknesses identified in the health system response	District health and local health staff, civil society representative of demographic and socioeconomic groups most affected
III. Analysis	
1. Complete draft framework based on analysis and synthesis of findings	Responsible personnel or task force from I(1) above, with assistance from health information/research staff
2. Prepare draft action plan, with built-in targets, indicators, and milestones	Responsible personnel or task force from I(1) above, and key persons responsible for eventual implementation
IV. Feedback to main stakeholders	
1. Provide feedback and obtain comments from stakeholders	A representative selection of key stakeholders involved in previous consultations and those who will be mainly affected by/involved in proposed changes
2. Share draft action plan and revise according to stakeholders’ input	As above
V. Implementation and monitoring of action plan	Health system representatives, clients and other stakeholders identified in Step IV above

## Paper context

A common finding of gender-related health research is that gender inequalities prevail in health systems worldwide. Existing tools to incorporate gender into health systems are complex and little used. New, simpler approaches, more intuitive for health workers and applicable to different health conditions and at different levels of the system, are needed. Our framework responds to this need, building on the growing awareness of human rights obligations in health and WHO’s health system building blocks.
